# Frontline Health Care Workers' (HCWs) perception of barriers to managing COVID-19 in Fiji

**DOI:** 10.3389/fpubh.2022.877624

**Published:** 2022-08-29

**Authors:** Anjana Deo, Masoud Mohammadnezhad

**Affiliations:** ^1^Research Office, Fiji Ministry of Health and Medical Services, Suva, Fiji; ^2^Faculty of Health Studies, School of Nursing and Healthcare Leadership, University of Bradford, Bradford, United Kingdom

**Keywords:** COVID-19 management, barriers, healthcare workers, qualitative study, Fiji

## Abstract

**Background:**

Health Care Workers (HCWs) are at higher risk of COVID-19 infection with their efforts while protecting the greater community and also exposed to hazards, such as psychological distress, fatigue, and stigma. This study aimed to explore the perception of frontline HCWs on barriers of managing COVID-19 in Fiji.

**Methods:**

A qualitative study method was approached to conduct this study among the HCWs who worked on the frontline during the COVID-19 pandemic based at ten purposively selected health facilities in the Suva subdivision in the Central Division of Fiji. The Colonial War Memorial Hospital (CWMH), the Fiji Centre for Communicable Diseases (Fiji CDC), Twomey hospital laboratory, and CWMH laboratory did the main control and most of the operations of other divisions were monitored from these settings. A semi-structured open-ended questionnaire was used to collect data using in-depth interviews. The participants' responses were audio-recorded and were later transcribed and analyzed using thematic analysis.

**Results:**

A total of twenty-nine HCWs took part in the in-depth interview and the responses were grouped into four themes, which include: workload, poor communication, lack of resources, and hindrance to education. It was also found through this study that some of the HCWs felt tired, frustrated, got rude to patients, and found it difficult to handle situations, which affected them mentally and physically stressed.

**Conclusion:**

Managing the COVID-19 cases has been attributed to the presence of many barriers, such as workload, tiredness, frustration, and sometimes difficult-to-handle situations, and the HCWs were indeed affected mentally and physically. Regular training for HCWs and more awareness programs would help the general public to follow the preventive measures, which reduces the cases and would help the HCWs manage COVID-19 well.

## Background

According to the Centers for Disease Control and Prevention (CDC), COVID-19 is a respiratory disease caused by severe acute respiratory syndrome-coronavirus 2 (SARS-CoV-2) (1). As of 12 February 2022, according to Johns Hopkins University, the number of global cases reached 408,553,932 and the number of deaths was 5,802,661 (2). The viruses constantly change through mutation and new variants of a virus are expected to occur. Some variants seem to spread more easily and quickly than other variants, which may lead to more cases of COVID-19. An increase in the number of cases will put more strain on healthcare resources, lead to more hospitalizations, and potentially more deaths (3).

For health and Health Care Workers (HCWs) around the world, the pandemic caused a heightened risk of occupational exposure to a new fast spreading disease and created the need to adapt roles and responsibilities for a wide range of tasks and professional settings. HCWs are not only at a high risk of infection, but they may also contribute to their spread from hospital to community environments and vice versa (4). This pandemic resulted in many infections and deaths among HCWs and their households (5). The health care sector is one of the most severely hit by the pandemic as the HCWs face multiple hazards that affect their physical, mental and social well-being (6). Lack of preparedness, the pandemic-related changes in working conditions, and access to and utilization of healthcare seemed to interact and create social tension among HCWs (7).

Hand hygiene has been one of the most important factors of infection control in a healthcare setting, according to a cross-sectional survey in Saudi Arabia. The hand hygiene knowledge and perception of the HCWs showed that the majority had moderate knowledge (57.8%) with perception (73.4%) of hand hygiene (8). The HCWs who were trained on hand hygiene were more likely to have good/ moderate perceptions than those without training. Another study in Bangladesh among dentists showed those with higher qualification levels had more knowledge and better practice toward COVID-19 than lower level qualifications, which indicated the more training for HCWs on infectious diseases the better knowledge and practice toward the pandemic (9).

According to the study, frontline COVID-19 healthcare workers were 52.0% more likely to have depression, 57.0% more likely to have anxiety, and 60.0% more likely to experience distress than non-frontline healthcare workers (10). The study on subjective burden and perspectives of German HCWs during the COVID-19 pandemic shows that there is a high increase of mental stress among the HCWs as worrying about their future regarding the well-being of their family and HCWs revealed that there is less time for personal life, and reduced in sleeping quality (11).

The COVID-19 pandemic has made psychological stress on HCWs in high-demand settings for long hours of work while they are separated from their families and facing social stigma (12). A review of health workers in China and Singapore found that one in four reported depression and anxiety, and one in three suffered from insomnia during COVID-19. The WHO also recently highlighted an alarming rise in reports of health workers being targets of verbal harassment, discrimination, and physical violence during this pandemic. In Fiji, the first case of COVID-19 was reported on the 19th of March 2020, followed by the second wave of Delta variant reported on the 26th of April 2021. While the international border has opened and the third wave of the outbreak began in December 2021 and currently the Omicron variant is being recorded in Fiji (13). Fiji has received the majority of the funds and medical equipment to assist in preventing and controlling COVID-19 for frontline HCWs as they play a vital role in stopping the virus in its tracks. On 28 August 2020, Fiji received Personal Protective Equipment (PPE) and other medical supplies from the Asian Development Bank and the Government of Japan which were procured by the United Nations Children's Fund (UNICEF), so that the frontline HCWs are well equipped, as well as protected from potential infections and enable the undisrupted delivery of essential services to the communities being served (14).

Most of the study shows that some of the attitudes among HCWs toward COVID-19 are that they are afraid of being infected and some are in fear of transmitting the disease to their families due to the unavailability of (PPEs). The study aimed to explore the barriers faced by frontline HCWs in managing COVID-19 in Fiji using a qualitative study.

## Materials and methods

### Study design and setting

An exploratory qualitative study design was conducted following the in-depth interviews among the HCWs on the perception of COVID-19 in Fiji from 1 August 2021 to 1 September 2021. The study was held at the Colonial War Memorial Hospital (CWMH), Ministry of Health and Medical Services (MHMSs), Fiji Centre for Communicable Diseases (Fiji CDC), St. Giles Hospital, Maternal Child Health Clinic—Namosi House, and Emergency Operations Team—Central/ Eastern, Twomey Hospital Lab, Samabula Health Centre, Makoi Health Centre, and Raiwaqa Health Centre. Since these settings were the main control for COVID-19 and most of the operations for COVID-19 were conducted in these settings, therefore, these settings were purposively chosen.

### Study sample

The participants for this study were doctors, nurses, health inspectors, and lab technicians from the ten health facilities in the Suva Central subdivision. The inclusion criteria were the following: the HCWs of both genders, all ethnicity groups, located in Suva subdivision health facilities, licensed under Fiji Medical Association and Fiji Nursing Council, and working as the frontline for COVID-19. Those HCWs not willing to participate in this study were excluded from this study. A total of twenty-nine HCWs were chosen purposively, and they were interviewed based at the health facilities and these included the following: 8 doctors, 8 nurses, 4 health inspectors, and 8 lab technicians located in Suva central division. The data was collected until the theoretical data saturation was achieved. Data saturation refers to the point in the research process when no new information is discovered in data analysis, and this redundancy signals to researchers that data collection may cease (15).

### Characteristics of the participants

The twenty-nine HCWs participated in this study were majorly women (72.4% and doctors (31%). Most of the participants were from the age group 30–39 years (38%). In terms of work experience, the majority were <5 years' experience (24.1%). Looking at the education level, the majority of the participants had studied up to Bachelor's degree (55.2%) (Table 1).

**Table 1:** Characteristics of HCW participants (*n* = 29).

**Characteristics**	**Descriptions**	**Frequency**	**Percentage**
Gender	Male	8	27.6
	Female	21	72.4
Age groups	20–29	6	20.7
	30–39	11	38
	40–49	10	34.5
	50 and above	2	6.9
Health care workers	Doctor	9	31
	Nurse	8	27.6
	Health Inspector	4	13.8
	Lab Technician	8	27.6
Years of experience as HCW	<5	7	24.1
	6–10	6	20.7
	11–14	3	10.3
	15–19	6	20.7
	20–24	3	10.3
	More than 24 years	4	13.8
Education level	Undergraduate diploma	1	3.4
	Bachelors	16	55.2
	Postgraduate certificate	1	3.4
	Postgraduate diploma	2	6.9
	Masters	9	31

### Data collection tools

A semi-structured, open-ended questionnaire was used as a guide to conduct in-depth virtual interviews, as in-depth interviews help individuals talk about their everyday experiences (16). Based on the literature review and the study research questions, the questionnaire was developed, which consisted of 20 questions. The questionnaire had 2 sections; demographics with 6 questions and 14 open-ended questions in the second section. The data collection was carried out in the English language as the responders are well qualified to answer the questions in English. The pretest was conducted on the five HCWs based on the prepared questionnaire and amendments were done where needed, while the timing was also measured during the pretest interview.

### Study procedure

The heads of the departments included the Divisional Medical Officer (DMO) of the Central division, the head of the Environmental Health Department in Namosi house, the head of the lab department in Fiji CDC and CWMH, and the Suva Sub-Divisional Medical Officer (SDMO) were approached to formally inform them about the study to seek their approvals to pursue with the research at the ten health centers in Suva. Followed by this, the HCWs located at the ten health centers were approached either by email or phone calls, where they were informed about this study a week and explained well before the proper interview was started. A verbal short introduction about this study was provided by the researcher to the HCWs on daily basis for the period of 1 month of the data collection. Together with this verbal introduction, an information sheet was provided to all the HCWs (who fulfilled the inclusion and exclusion criteria). Those HCWs who agreed to participate in this study were given the consent form. The virtual interview was carried out by the principal investigator, with those HCWs who agreed to be part of this study. All interviews were recorded which lasted for ~30–40 min each.

### Data management and analysis

All the interview recordings were transcribed verbatim by the principal researcher. Transcription was done on the same day of the interview. A review of transcriptions was done to correct errors and to remove references of names and places to ensure anonymity for the participants. Once the transcriptions were clarified, data analysis was carried out.

Manual thematic analysis was used to analyze the data in this study. Thematic analysis is a method for identifying, analyzing, organizing, describing, and reporting themes found within a data set. Thematic analysis was carried out using four steps that is immersion in the data, coding the data, creating categories, and identifying themes/subthemes (17).

The principal researcher read and re-read all interview transcripts and identified similar phrases and words for which numbers were assigned. The coded data that had similar characteristics were grouped together. Once the grouping of similar data was completed, descriptive themes and sub-themes were identified to reflect the perceptions of participants (18).

### Study rigor

In qualitative research, it is vital to establish and increase rigor and trustworthiness of qualitative (19). Four criteria were identified that contribute to trustworthiness. These criteria consist of credibility, transferability, dependability, and confirmability (20).

To ensure credibility, all study participants were actively involved in the interview process. They were asked to clarify if more information was needed during the interviews. To make sure the interview process was correct, it was continuously discussed with the co-researcher. To ensure dependability, all in-depth interview questions asked to HCWs were the same. All interviews were transcribed independently and coded and themed by the research team. Transferability was ensured using purposive sampling, discussing study methodology, and reaching data saturation. Study conformity was ensured by recording the interviews and taking notes.

### Ethical considerations

The ethical approval was obtained from the College Health Research Ethics Committee (CHREC) of Fiji National University (FNU) with ID# 305.20 and the Fiji Human Health Research Ethics Review Committee (FHHRERC) at MHMSs, which was further approved by the Permanent Secretary of MHMS as this is related to the current crisis.

All participants were given the information sheet at the beginning and were asked to sign the consent form. All the participants were informed that their participation in this study was totally voluntary and they could leave the study at any stage they wanted to, and also all the information was kept confidential and their names were anonymous.

## Results

### Themes identified

**Figure 1:**
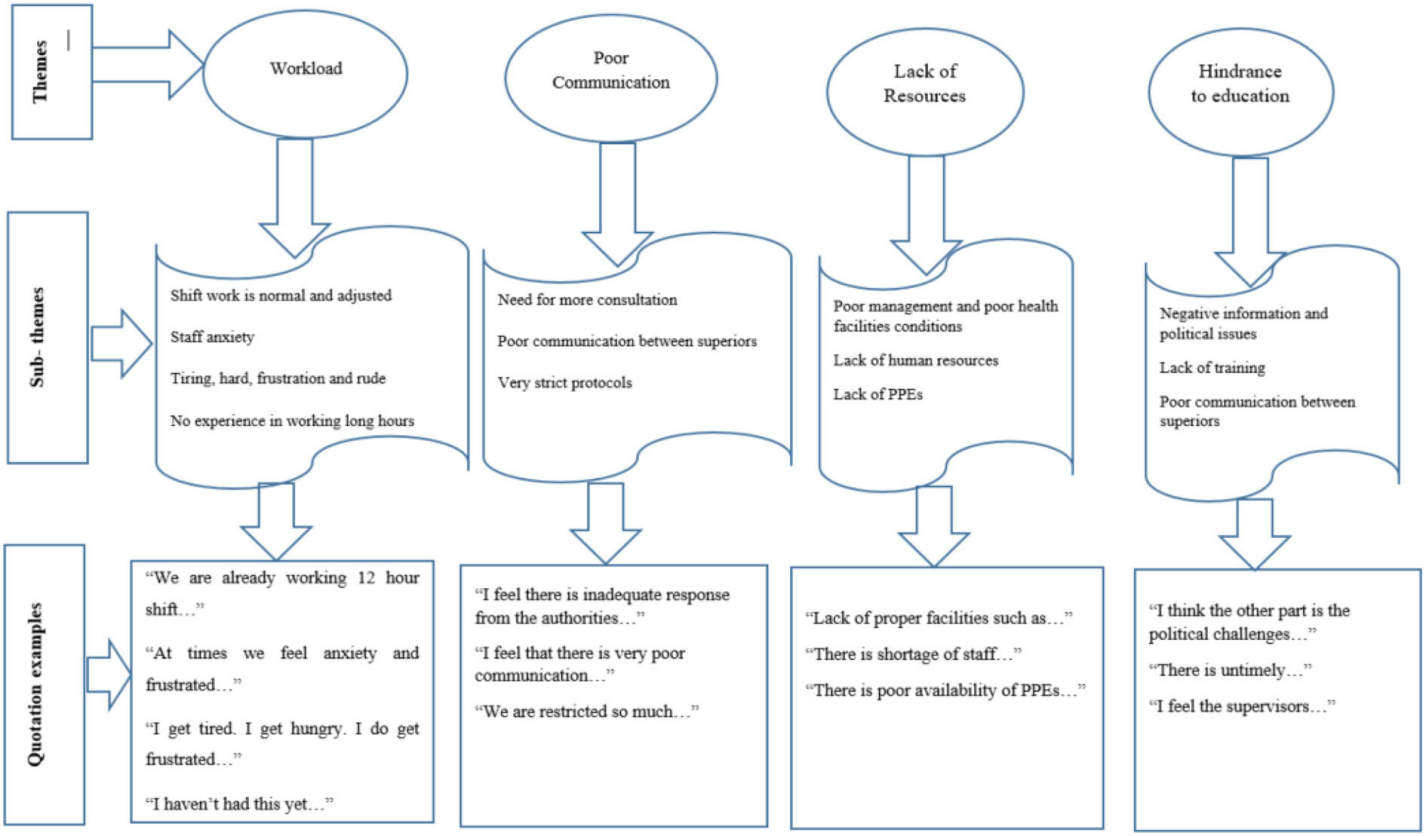
Themes identified with open codes.

Four themes emerged from the data analysis: workload, poor communication, lack of resources, and hindrance to education. Figure 1 summarizes the themes and codes. In the result section, to protect the privacy of participants, their specialists and numbers were used for the quotations. For the Doctors we used—(D1, D2 …); (N1, N2 …) was used for Nurses; we used (HI1, HI2 …) was used for the Health Inspectors; and for the Laboratory Technicians we used (LT1, LT2 …).

#### Theme 1: Workload

Few questions were asked about workload during COVID-19 in terms of treating the patients or handling the swabs and other issues. Below describes the burden effects on COVID-19.

#### Burden effects on COVID-19

The HCWs were mentioned as normal and adjusted to the shift work while managing the COVID-19 situation.

“*We are already working 12 hour shifts before pre pandemic times so it's not really much of the difference for any emergency doctor, 12 hour shift is a norm for us, so not much of a change.”* (D9, a 35-year-old male)

The HCWs mentioned as staff anxiety and staff frustration issues while managing the COVID-19 situation.

“*At times we feel anxiety and frustrated while we are loaded with lots of work.”* (D1, a 40-year-old female)

Some of them mentioned tiredness, harshness, frustration and rudeness, and mental and physical effects while managing the COVID-19 situation.

“*I get tired. I get hungry. I do get frustrated easily then if things don't go as planned. There is no option but to manage it. I try to eat when I can. I pray every day that situation will be okay. I feel the staffs feel anxiety, mental health issues.”* (D4, a 26-year-old male)

They also mentioned as no experience in working long hours while managing the COVID-19 situation.

“*I haven't had this yet, but it must be tiring but a sacrifice worth making given the situation we're in.”* (D2, a 30-year-old female)

#### Theme 2: Poor communication

Few questions were asked about communication during COVID-19 in terms of treating the patients or handling the swabs and other issues. Below describes the consultations.

#### Consultations

The HCWs mentioned that there was a need for consultation on guidelines and more awareness in different languages while managing the COVID-19 situation.

“*I feel there is inadequate response from the authorities, not enough consultancy on SOPs, not enough trainings or awareness to the health staff, there is also poor risk communication to staff and public.”* (D1, a 40-year-old female)

Some of them mentioned that there was poor communication among the HCWs while managing the COVID-19 situation.

“*I feel that there is very poor communication between the superiors, as some are not sharing the information with their colleagues*.” (D7, a 28-year-old male)

They also mentioned that there were very strict protocols while managing the COVID-19 situation.

“*We are restricted so much, one of the challenge is trying to update family, it is important that they know, what is happening because they are here with us and the families at home, because of restrictions, curfews and one of the challenges is trying to relay the messages. You will have may be 30 patients and you have to call almost everyone and you have to have credit in your official phone to do this. They have to have patience to relay messages and most time not a good messages because they are getting unwell and providing them with because we care after when they go home and continue rehabilitation*.” (D8, a 42-year-old female)

#### Theme 3: Lack of resources

Few questions were asked on resources while managing COVID-19 in terms of treating the patients or handling the swabs and other issues. Below describes the management, health facilities, and financial issues.

#### Management, health facilities, and financial issues

The HCWs mentioned that there were poor management, poor isolation rooms, conditions, and spacing in health facilities while managing the COVID-19 situation.

“*Lack of proper facilities such as toilet facilities, hot and cold system, disposable paper towels, tissue or toilet paper, hand soap etc.”* (HI3, a 35-year-old female)

They also highlighted that there was a lack of human resources and other lack of medical resources available while managing the COVID-19 situation.

“*There is shortage of staff and increase in the swab samples. The supporting equipment not provided together with testing analysers such as AC not upgraded or even given a new one, existing, as playing up due to heat generated from new analyser.”* (LT5, a 30-year-old female)

Some HWCs highlighted specifically on lack of PPEs while managing the COVID-19 situation.

“*There is poor availability of PPEs in the health facilities.”* (D4, a 26-year-old male)

#### Theme 4: Hindrance to education

Few questions were asked about communication during COVID-19 in terms of treating the patients or handling the swabs and other issues. Below describes the negative impact on education.

#### Negative impact on education

The HCWs mentioned that there were negative information, political issues, and language barriers while managing the COVID-19 situation.

“*I think the other part is the political challenges, there are certain agendas that are put from a political perspective versus a technical perspective, so those challenges that we have to mitigate and manage within. Other than that some resistance from public and people in terms of the responses that we are putting out the non-hindrance to covid safe behaviors like those that are actually continuing to breach certain protocols curfew breaches or looking at gatherings playing sports or those that are continue to drink kava and funeral gatherings, those sort of things I consider a challenge.”* (HI4, a 42-year-old male)

They also highlighted that there were inadequate training, awareness, and COVID-19 SOPs were not available while managing the COVID-19 situation.

“*There is untimely SOPs and not enough training and awareness.”* (D1, a 40-year-old female)

Some of the HCWs highlighted that there was little sharing of knowledge by the superiors while managing the COVID-19 situation.

“*I feel the supervisors are not communicating well with their staff, in terms of sharing information or any knowledge they obtain lately.”* (D7, a 28-year-old male)

## Discussion

While managing the COVID-19 situation, the HCWs adjusted the workload, as they worked as a team, with a trustful, friendly, and supportive environment. Some of the HCWs felt tired, frustrated, got rude to patients, found it difficult to handle situations, were affected mentally and physically, and were stressed. Few of the HCWs did not have experience working long hours. A study shows due to inadequate quantities of PPEs, developed anxiety and apprehension of the fear of infection among the HCWs (21). It also showed that long working hours for HCWs during the COVID-19 outbreak had contributed as a risk factor to COVID-19 infection among HCWs. A survey in Italy showed that the HCWs' perception of the risk of being infected was significantly higher in the workplace than in the outside environment (22).

Moreover, in terms of poor communication, the HCWs mentioned that there was a need for consultation on guidelines and more awareness in different languages while managing the COVID-19 situation. The HCWs also highlighted that there was poor communication within the HCWs while managing the COVID-19 situation. Some of the HCWs mentioned that the COVID-19 protocols were very strict, this was in terms of the COVID-19 restrictions and curfews, making it hard to contact the patient's families. A study in Nepal shows that 61% of health workers had poor knowledge about COVID-19 transmission (23). Additionally, a study shows almost 50% of respondents were unsure or did not know to whom and to where COVID-19 cases should be reported. Similarly, the majority of HCWs did not participate in educational activities and emergency planning for COVID-19 (24).

Furthermore, in terms of the lack of resources, the HCWs mentioned that there were poor management, poor isolation rooms, conditions, and spacing in health facilities. They also felt that there was a lack of human resources and other lack of medical resources available while managing the COVID-19 situation. The HCWs mentioned that there were miscommunication, political issues, and language barriers while managing COVID-19. A study in Australia shows that the HCWs were more concerned about their job security and many reported lost work hours (25). Another study in Oman reveals that the challenges are linked to the insufficient infectious disease and public health expertise at the field level and the intensive workload that drained HCWs physically and emotionally (26).

Moreover, a study shows that the most common barrier was the lack of awareness among the general population about COVID-19 preventive measures (89.1%). Other common perceived barriers included poor healthcare infrastructure (85.2%), an insufficient supply of PPE (86%), lack of affordable hand sanitizers and facemasks for the public (83.9%), and inadequate financial resources for COVID-19 prevention and control (82.7%). Half of the participants perceived the weak performance of the local media in spreading awareness of COVID-19 as a barrier. The lack of training on infectious disease outbreaks and the absence of emergency response protocols were also perceived as barriers by 67.7 and 72.2% of respondents, respectively (24).

### Limitations

It would be ideal for future researchers to include all the HCWs who were involved in managing COVID-19 and those who were present in health facilities and also all the community health workers. Due to the setting of the study and the nature of the study participants, the timing of data collection was limited. Data was collected based on the time available for the participants as it was collected during the peak situation of the second wave of the outbreak in Fiji. The study was conducted only in the health facilities in Suva, which were mostly situated in urban areas, and hence leaving out the health facilities which were situated in rural areas that might have different perceptions about COVID-19.

## Conclusion

The HCWs had mentioned that all the COVID-19 precautionary measures were practiced at the health facility when the patients or visitors visited the health facility, while the HCWs also practiced all the precautionary measures while managing the patients. Virtual communications were also practiced for any form of training and meetings as a COVID-19 precautionary measure. In terms of the COVID-19 precautionary measures outside the health facility, such as masks, hand hygiene, social distancing, and isolation, the HCWs practiced all these, however, sometimes they forget especially the mask-wearing. All the training was practiced virtually due to the COVID-19 precautionary measures and HCWs do not have any issues with this.

In conclusion, the HCWs had knowledge on COVID-19 and they practiced all the COVID-19 restrictions while managing the cases in Fiji. This had been attributed to the presence of many barriers, such as workload, tiredness, frustration, and sometimes difficult-to-handle situations, getting the HCWs affected mentally and physically. The HCWs highlighted the lack of awareness on COVID-19 preventive measures to the general public, as awareness programs would help in the reduction of the cases and helps the HCWs in managing the COVID-19 cases well.

## Data availability statement

The raw data supporting the conclusions of this article will be made available by the authors, without undue reservation.

## Ethics statement

The studies involving human participants were reviewed and approved by College Health Research Ethics Committee (CHREC) of Fiji National University. The patients/participants provided their written informed consent to participate in this study.

## Author contributions

AD: conceived and designed the experiments, performed the experiments, analyzed and interpreted the data, contributed reagents, materials, analysis tools or data, and wrote the paper. MM: conceived and designed the experiments, analyzed and interpreted the data, contributed reagents, materials, analysis tools or data, and wrote the paper. Both authors contributed to the article and approved the submitted version.

## Conflict of interest

The authors declare that the research was conducted in the absence of any commercial or financial relationships that could be construed as a potential conflict of interest.

## Publisher's note

All claims expressed in this article are solely those of the authors and do not necessarily represent those of their affiliated organizations, or those of the publisher, the editors and the reviewers. Any product that may be evaluated in this article, or claim that may be made by its manufacturer, is not guaranteed or endorsed by the publisher.
